# Binaural consequences of speech envelope enhancement

**DOI:** 10.1121/10.0015155

**Published:** 2022-11-15

**Authors:** Lucas S. Baltzell, Daniel Cardosi, Jayaganesh Swaminathan, Virginia Best

**Affiliations:** Department of Speech, Language, and Hearing Sciences, Boston University, Boston, Massachusetts 02215, USA lbaltzel@bu.edu, dcardosi@bu.edu, jswamy@bu.edu, ginbest@bu.edu

## Abstract

The potential binaural consequences of two envelope-based speech enhancement strategies (broadband compression and expansion) were examined. Sensitivity to interaural time differences imposed on four single-word stimuli was measured in listeners with normal hearing and sensorineural hearing loss. While there were no consistent effects of compression or expansion across all words, some potentially interesting word-specific effects were observed.

## 1. Introduction

The perceptual importance of modulations in speech has long been recognized. In quiet, spectrally reduced speech can be understood primarily on the basis of temporal information [e.g., Shannon *et al.* (1995)], and there is a long history of experimental and modeling work relating the changes to speech modulations introduced by noise or other kinds of environmental interference to degraded intelligibility [e.g., Steeneken and Houtgast (1980), Payton and Braida (1999), Jørgensen and Dau (2011), and Houtgast *et al.* (2002)].

Following this logic, a number of algorithms have been developed to enhance the salience of speech modulations with the goal of improving intelligibility under certain listening conditions. Some of these algorithms were developed with cochlear implants in mind, where the intelligibility of the speech signal depends entirely on the modulations described by the envelope. Using a noise vocoder to process stimuli, Lorenzi *et al.* (1999) showed that for speech in quiet, squaring the envelope signal resulted in a slight decrease in intelligibility, while for speech in noise, squaring the envelope resulted in a slight increase in intelligibility [see also Fu and Shannon (1998)]. A number of studies have since modified/refined this “envelope-expanding” approach with somewhat mixed results [e.g., Apoux *et al.* (2001), Apoux *et al.* (2004), and Narne and Vanaja (2009)].

A different class of algorithms, which we refer to here as “envelope-compressing” algorithms, have also been developed to improve speech perception. Vandali (2001) developed an algorithm that identifies and amplifies short-duration bursts of energy. Since these short-duration bursts tend to correspond to consonants, and since consonants are typically lower intensity compared to vowels, this algorithm effectively compresses the broadband envelope [see also Skowronski and Harris (2006)]. Desloge *et al.* (2017) developed a broadband envelope compression algorithm designed to improve speech intelligibility in the presence of fluctuating noise. In this case the goal was not to manipulate the existing modulations in speech but to exploit slower fluctuations in signal-to-noise ratio (SNR). By boosting the intensity of low energy portions of the signal, which tend to be speech, they showed improved intelligibility for a group of listeners with hearing loss [see also D'Aquila *et al.* (2017) and Goldsworthy *et al.* (2022)]. It is worth noting that the non-linear compression that is present in nearly all modern hearing aids acts as an envelope compressor, though compression is typically applied within frequency bands and with a variety of considerations (such as correcting for loudness recruitment) in mind (Dillon, 2012).

Common to both expansive and compressive approaches is the goal of improving speech intelligibility, in quiet or in noise. Our observation, however, is that these approaches have been evaluated exclusively under monaural conditions, in which binaural cues are not relevant. The binaural consequences of these strategies have not previously been investigated, despite good reasons to believe that modifications of the envelope might influence the salience of binaural cues. In particular, it has long been recognized that abrupt rises in the amplitude envelope (or “onsets”) have special significance for the coding of binaural information, and this phenomenon has received renewed attention in recent years as researchers have tried to characterize binaural “onset dominance” for temporally complex sounds [for review, see Stecker *et al.* (2021)]. Of particular relevance here, interaural time differences (ITDs) occurring at onsets in the envelope tend to dominate the overall spatial percept of modulated sounds, and the steepness of these rising portions of the envelope is known to affect the salience of ITDs (Klein-Hennig *et al.*, 2011; Laback *et al.*, 2011; Dietz *et al.*, 2013; Stecker, 2018). This also appears to be largely true for speech (Baltzell *et al.*, 2020; Baltzell and Best, 2021), though exactly what spectro-temporal features constitute a perceptually robust onset for speech stimuli has not been determined. It is on these bases that we might expect binaural consequences of different envelope enhancement algorithms.

A number of algorithms have been developed that manipulate the speech envelope with the explicit goal of enhancing onsets. Koning and Wouters (2012, 2016) describe an algorithm that emphasizes transient onsets in the signal by subtracting a high-pass copy of the envelope from a low-pass copy. The goal of this manipulation is to increase release from neural adaptation, which occurs in auditory nerve fibers in response to sustained stimulation. Release from adaptation is also thought to contribute to binaural onset dominance, which has motivated at least one algorithm designed to increase the salience of binaural cues. Monaghan and Seeber (2016) describe an envelope sharpening algorithm designed to increase the steepness of onsets in the stimulus. In listeners with normal hearing given simulated cochlear-implant signals (sine-wave vocoded speech with diotic carriers), they showed that the algorithm both improved ITD thresholds and increased extents of laterality.

The goal of the present study was to investigate the binaural consequences of broadband envelope expansion and compression for speech stimuli. In noisy or multi-talker environments, the ability to make use of spatial cues can yield a significant improvement in speech intelligibility [for review, see Culling and Lavandier (2021)], so any detrimental or beneficial effect of envelope-based speech enhancement strategies on binaural hearing should be considered. Of particular interest, however, was to identify potential improvements in binaural salience afforded by either strategy. The presence of such improvements would suggest additional benefits of these speech enhancement strategies that have so far been overlooked because they have only been evaluated under monaural listening conditions.

**Fig. 1.**
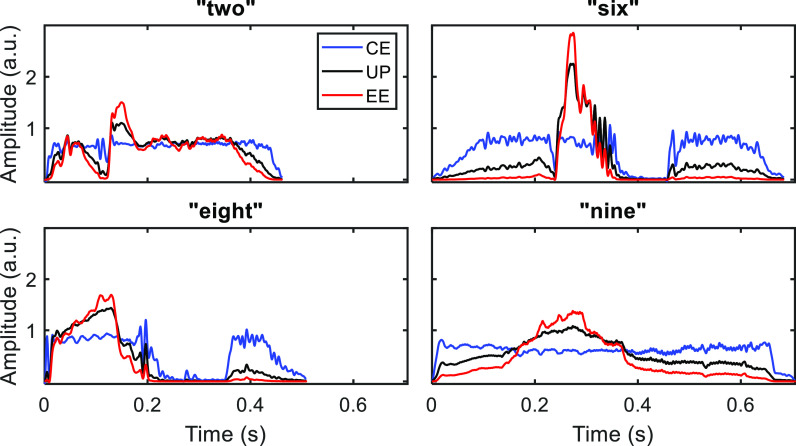
Envelopes for each word and for each processing condition. Envelopes were extracted for display by low-pass filtering (fourth-order, 128 Hz cutoff) the magnitude of the analytic signal, and are presented in arbitrary magnitude units (a.u.).

We measured ITD sensitivity for spoken words that were unprocessed (UP), envelope expanded (EE), or envelope compressed (CE). These manipulations were applied to the broadband envelope (Fig. 1). We considered three different listening conditions: speech in quiet, speech in multi-talker babble, and noise-vocoded speech with interaurally decorrelated carriers. The goal of interaural decorrelation was to limit access to ITDs contained in the temporal fine structure (TFS) to reveal potential effects related to ITDs carried in the envelope. In addition, we tested listeners with sensorineural hearing loss, who have reduced access to ITDs carried in the TFS and may place more perceptual weight on the envelope (Lacher-Fougère and Demany, 2005; King *et al.*, 2014).

## 2. Methods

### 2.1 Participants

There were 18 participants in total. This included nine listeners with audiometrically normal hearing (NH) (ages 19–43 years; mean age 25) and nine listeners with sensorineural hearing impairment (HI) (ages 18–47 years; mean age 29). In the HI group, hearing losses were all bilateral and symmetric but varied in severity and configuration, with pure-tone averages (0.5, 1, 2 kHz) between 15 and 61 dB. Three of the nine NH participants were authors of this paper, and the remaining NH and HI participants were recruited from the Boston University student population or the local community. All had some amount of experience with psychoacoustic testing. All procedures were reviewed and approved by the Boston University Institutional Review Board.

### 2.2 Stimuli

The stimuli were four speech tokens (“two,” “six,” “eight,” and “nine”) spoken by a female talker [and used previously by Baltzell and Best (2021)]. All stimuli were normalized to a presentation level of 60 dB SPL subsequent to processing. Additionally, for HI participants, linear frequency-specific gain according to the NAL-RP prescription formula was applied to ensure adequate audibility.

The word tokens were manipulated so that the broadband envelope was either compressed or expanded. The compression algorithm followed the basic structure of Desloge *et al.* (2017) but was applied to individual word tokens instead of sentence-length speech. Short-term and long-term time windows were defined, and each short-term window was multiplied by a gain factor inversely proportional to the magnitude (square root of energy) in that window compared to the window with the maximum magnitude within the long-term window

$$G_{\mathrm{compress}}\left[n\right]=\sqrt{\text{max}\left(E\left[:\right]\right)/E\left[n\right]}.$$ (1)

The goal of this gain factor was to reduce the variation in energy across short-term windows. Since all of the time bins had the same length, energy $E\left[n\right]$ in a particular window (i.e., the sum of squared time-domain amplitudes in that window) is proportional to power here. Short-term windows were 10-ms long with a 50% overlap. For each word, the long-term window duration was the total duration of that word. Following Desloge *et al.* (2017), the gain factor (when expressed in dB) was limited to a maximum of 20 dB. This means that the maximum gain that can be applied to low-intensity windows is 20 dB, which prevents over-amplification of windows near or at the noise floor.

The expansion algorithm was realized in the same framework, but the gain factor was set to be proportional (rather than inversely proportional) to the magnitude (square-root of energy) in a short-term window relative to the window with the maximum magnitude

$$G_{\mathrm{expand}}\left[n\right]=\sqrt{E\left[n\right]/\text{max}\left(E\left[:\right]\right)}.$$ (2)

Consistent with Lorenzi *et al.* (1999), no range limitation was applied, so in effect the energy in each bin was multiplied by itself. This procedure is conceptually equivalent to squaring the envelope.

The effects of compressing and expanding the envelopes of the four word tokens are shown in Fig. 1. After level normalization, compression has the effect of enhancing low-energy consonants (e.g., the word-initial consonants in “two,” “six,” “nine”), while expansion has the effect of enhancing high-energy vowels (e.g., the central vowels in “six,” “nine”).

The stimuli were presented under three different listening conditions. Both NH and HI groups completed a Quiet condition, in which the words were presented in isolation. NH listeners also completed a Babble condition, in which the words were embedded in a continuous multi-talker babble at an SNR of −5 dB. The babble was comprised of four streams of concatenated sentences from four different talkers (two male, two female). Random segments of the four-talker babble were drawn independently for each ear to create dichotic multi-talker babble. NH listeners also completed a Decor condition, in which the words were noise-vocoded and the carriers were interaurally decorrelated [following Baltzell *et al.* (2020), correlation value *r *=* *0.5] and presented in isolation.

### 2.3 Procedure

Stimuli were presented via Sennheiser HD 280 headphones (Wedemark, Germany) to listeners seated in a double-walled sound-attenuating chamber (IAC Acoustics, North Aurora, IL). The digital signals were generated on a PC outside of the booth and then routed through an RME HDSP 9632 24-bit soundcard (Haimhausen, Germany). Stimulus presentation level was normalized based on headphone calibration to a flat-spectrum broadband noise, and inverse filtering based on the headphone frequency response was applied. Headphone calibration was conducted using a Brüel & Kjær type 4143 artificial ear coupler (Virum, Denmark).

ITD thresholds were obtained for each of the four words (“two,” “six,” “eight,” “nine”) under each of the three processing conditions (UP, EE, CE) Thresholds were obtained in blocks for each processing condition, where each block contained 12 tracks for NH listeners (four words × three listening conditions) and 4 tracks for HI listeners (four words × one listening condition). Each block was presented twice, and conditions were randomized within each block for each listener. The order of blocks was also randomized for each listener for the first presentation, and this order was reversed for the second presentation.

ITD thresholds were measured in a two-alternative forced-choice lateralization task. On each trial, the word token was presented in two intervals, separated by a 500-ms inter-stimulus interval. In the first interval, the ITD was always 0 *μ*s and served as a reference for the second interval. In the second interval, the ITD was either left-leading or right-leading, and the listener was instructed to indicate whether the word token in the second interval was presented from the left or right of midline. The ITD was adaptively varied according to a three-down/one-up tracking procedure in log_10_ steps (targeting approximately 80% correct). ITDs were initially varied in step sizes of 0.2 log_10_ units and subsequently in step sizes of 0.1 log_10_ units after the fourth reversal. Each track consisted of 80 trials and began with an ITD of 100 *μ*s. Correct answer feedback was provided during testing. For each listener, data were pooled across the two tracks completed for each word in each condition, and psychometric functions were fit using the “psignifit” package (Schütt *et al.*, 2016). Thresholds were obtained by extracting ITD values corresponding to 75% correct on this function.

### 2.4 Results

**Fig. 2.**
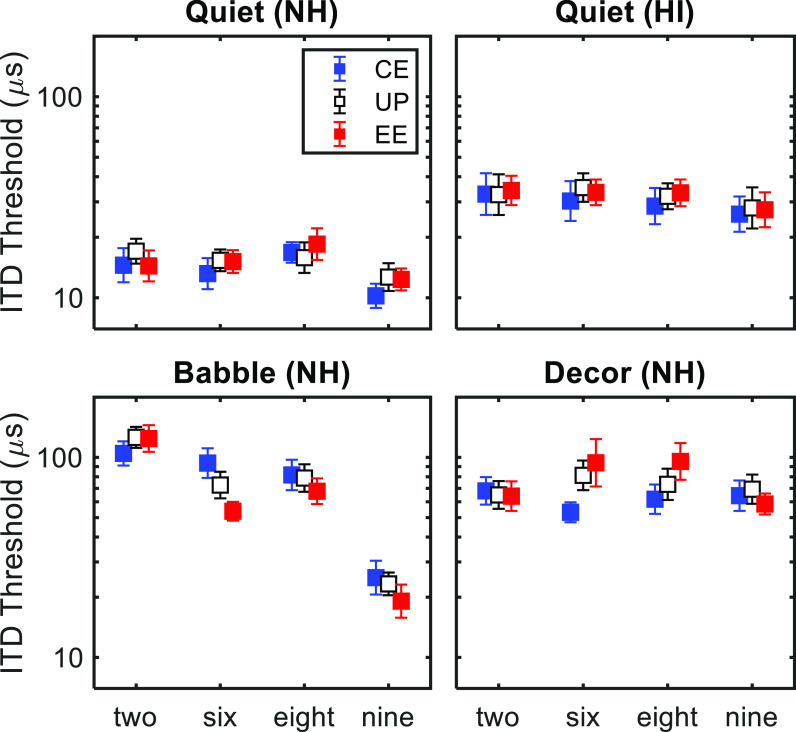
ITD thresholds for each listening condition, word, and processing condition. Symbols and error bars depict across-subject means and standard errors. For both the NH and HI groups, n = 9.

Results are shown in Fig. 2. A comparison of the two upper panels shows that HI listeners had higher thresholds overall, consistent with previous studies using similar stimuli (Best and Swaminathan, 2019; Baltzell *et al.*, 2020). For NH listeners, thresholds were also higher for the Babble and Decor conditions (lower panels) than in Quiet, as expected. Within each panel, there appear to be some consistent effects of word for NH listeners, especially in the Babble condition, where thresholds are clearly lower for “nine.” Compared to these larger effects, effects of processing condition are small and not observed consistently across words and conditions.

Because the NH and HI groups completed a different set of conditions, we conducted statistical analyses for each group separately. For the NH group, a mixed model was implemented using the “fitlme” function in matlab, treating listening condition, word, and processing condition as fixed effects and subject as a random effect (random intercept). Because this analysis revealed a significant three-way interaction between word, listening condition, and processing condition [*F*(12,280) = 1.88, *p *=* *0.037], we ran a set of *post hoc* ANOVAs for each listening condition (Table 1). We were primarily interested in the effect of processing condition, which was not significant for any listening condition. However, the interaction between processing condition and word was significant in both the Babble and Decor listening conditions, suggesting that there were effects of processing condition that were not consistent across words. For the HI group, a single ANOVA conducted on the Quiet data revealed no significant effects (Table 2).

**Table 1.** Results of *post hoc* ANOVAs conducted separately for each listening condition for the NH group.

	Quiet		Babble		Decor	
	*F*-statistic	*p*-value	*F*-statistic	*p*-value	*F*-statistic	*p*-value
Processing	*F*(2,88) = 1.4	*p *=* *0.253	*F*(2,88) = 1.38	*p *=* *0.256	*F*(2,88) = 0.07	*p *=* *0.931
Word	*F*(3,88) = 2.46	*p *=* *0.068	*F*(3,88) = 66.6	*p *<* *0.001	*F*(3,88) = 0.65	*p *=* *0.587
Processing^*^Word	*F*(6,88) = 1.01	*p *=* *0.427	*F*(6,88) = 2.95	*p *=* *0.011	*F*(6,88) = 2.26	*p *=* *0.045

**Table 2.** Result of ANOVA conducted for the HI group.

	Quiet	
	*F*-statistic	*p*-value
Processing	*F*(2,88) = 0.91	*p *=* *0.913
Word	*F*(3,88) = 1.24	*p *=* *0.301
Processing^*^Word	*F*(6,88) = 0.21	*p *=* *0.972

## 3. Discussion

The goal of this study was to reveal any effects of two broadband speech enhancement strategies (EE and CE) on ITD sensitivity as measured with single-word stimuli. Overall, we did not find consistent positive or negative effects of either strategy on ITD thresholds (i.e., effects that were consistent across the four different speech tokens we investigated) in any condition. Despite this failure to find consistent effects across words, there was some evidence for word-dependent effects of EE and CE in the more challenging listening conditions. A closer inspection of the data in Fig. 2 suggests that for the Babble condition, EE tended to improve thresholds, while CE had both positive and negative effects depending on the word. Conversely, in the Decor condition, CE tended to improve thresholds, while EE had a mix of positive and negative effects. The variations in the presence and size of these effects across words likely reflects the fact that EE and CE emphasize certain features of certain words (Fig. 1) and also that listeners place their weight on different parts of the envelope for different words (Baltzell and Best, 2021).

Of course, the findings presented here are limited to the specific algorithms we considered, which are complementary but do not represent the full range of algorithms that have been developed in this domain. It is possible that different algorithms or parameter choices could have resulted in larger or more consistent effects on ITD sensitivity. In particular, the algorithms we tested operated on the broadband speech envelope, whereas the within-channel envelope is likely the relevant feature for ITD sensitivity. Future extensions of this work will investigate the effect of within-channel EE and CE strategies on binaural perception.

Whether using a within-band or broadband approach, it would be interesting to probe the effects of CE and EE for a wider variety of speech sounds to reveal any generalized benefits. Our results suggest that further testing of EE in the presence of interfering sounds as well as further testing of CE under conditions where TFS is unreliable (such as in reverberation) may be fruitful. It would also be interesting to extend these investigations to other tasks that rely on suprathreshold ITDs.
